# Supplementary material for: Assessment of Spinal Muscular Atrophy Carrier Status by Determining SMN1 Copy Number Using Dried Blood Spots
Source: Int J Neonatal Screen. 2020 May 29;6(2):43. doi: 10.3390/ijns6020043 (PMC7423012; doi:10.3390/ijns6020043)
Supplement: Supplementary file 1 [file IJNS-06-00043-s001.pdf]

Supplementary Material

Detection of homozygous *SMN1* deletion in the DBS samples on Guthrie cards

We have reported an *SMN1* deletion test using a combination method of targeted pre-amplification (first round PCR) and mCOP amplification (second round PCR) to detect *SMN1* [13, 18]. The starting material in the previous study was DBS on FTA cards. Here, we obtained *SMN1* deletion test results using Guthrie cards, instead of FTA cards.

1. Mongolian and Indonesian patients with SMA

The *SMN1* amplification curves of Patients 1, 2, 3 and 4 showed no rise before 16 cycles in the second round PCR, while those of controls rose at a steep rate at ~10 cycles. However, the *SMN2* amplification curves of Patients 1, 2, 3, and 4 showed steep rises at ~10 cycles in the second round PCR, similar to controls. We show the amplification curves and melting peak analysis of Patient 1, which represents results for Patients 1, 2, 3 and 4 because all samples produced very similar results (Figure S1). These results indicated that these patients carried homozygous *SMN1* deletions, confirming the diagnosis of SMA.

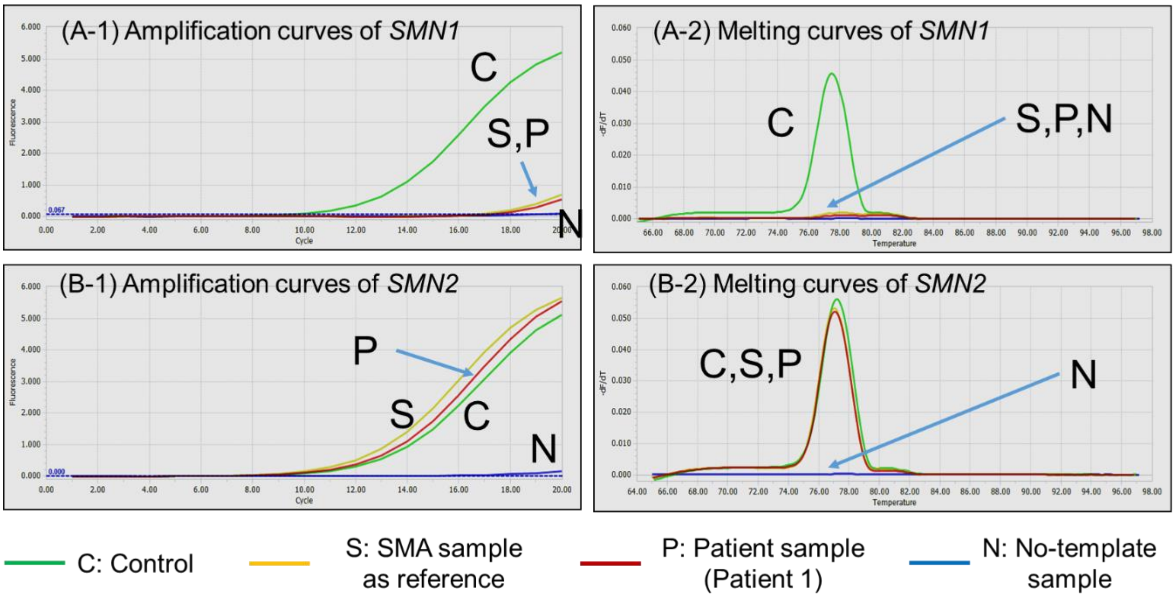

Figure S1. Amplification and melting curve analysis for Patient 1. Amplification and melting curves of *SMN1* and *SMN2* for Patient 1 (P, red line), Control (C, green line), SMA reference (S, yellow line) and No-template (N, blue line) samples are shown. Patient 1 showed no amplification of *SMN1*, but significant amplification of *SMN2*.

2. Parents of SMA Patients

The *SMN1* and *SMN2* amplification curves of the parents of Patient 1 and 4 showed steep rises at ~10 cycles in the second round PCR, which was the same as controls. We show the amplification curves and melting peak analysis of Patient 1's father, which are representative of all parents (Figure S2) These data showed that the father and mother were not affected by SMA, but the data did not indicate whether they were SMA carriers or not. To determine this, it is necessary to determine the *SMN1* copy number.

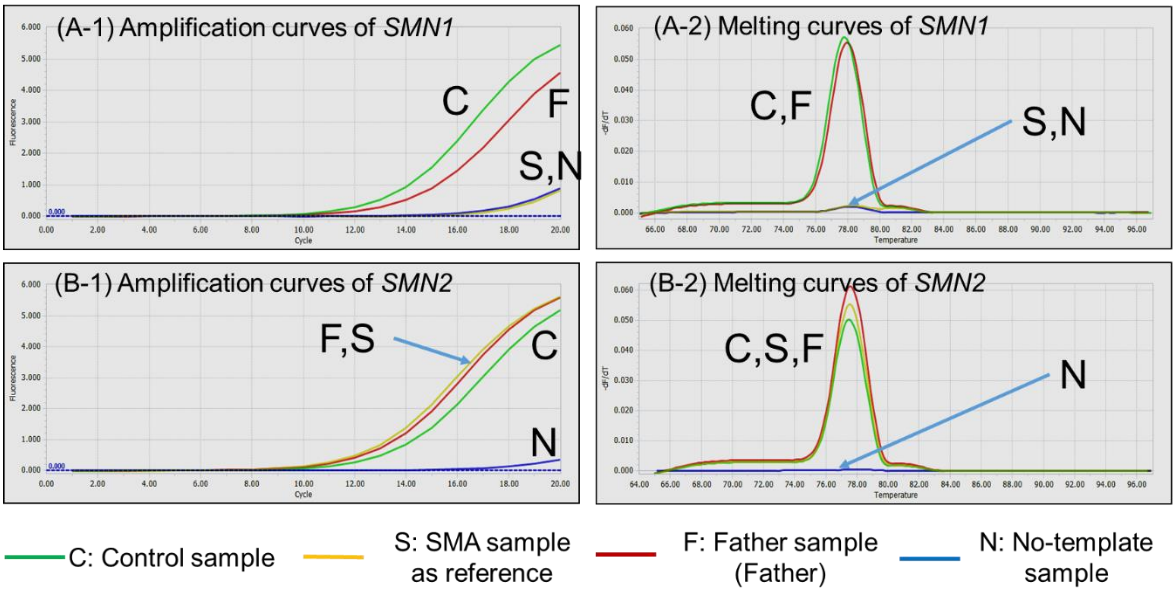

Figure S2. Amplification and melting curve analysis of Patient 1's Father. Amplification and melting curves of *SMN1* and *SMN2* of the Father (F, red line), Control (C, green line), SMA reference (S, yellow line) and No-template (N, blue line) samples are shown. The father showed significant amplification of *SMN1* and *SMN2*.
